# THE SOCIOCULTURAL STRESS PROCESS MODEL APPLIED TO LONG-DISTANCE CAREGIVING

**DOI:** 10.1093/geroni/igz038.2057

**Published:** 2019-11-08

**Authors:** Verena R Cimarolli, Amy Horowitz, Danielle Jimenez, Xiaomei Shi, Francesca Falzarano, Jillian Minahan

**Affiliations:** 1 LeadingAge LTSS Center @UMass Boston, Washington, District of Columbia, United States; 2 Fordham University, New York, New York, United States; 3 LeadingAge LTSS Center @UMass Boston, Boston, Massachusetts, United States

## Abstract

This study investigated the impact of LDC on mental health utilizing the Sociocultural Stress Process Model as a conceptual framework. A path analytic model tested the impact of caregiving stressors (i.e. distance, frequencies of visits, hours spent helping, burden) and sociocultural values (i.e. familialism) on LDCs’ mental health outcomes (i.e. depression, anxiety), and resources (i.e. coping strategies, social support) which can mediate the association between stressors and mental health outcomes while controlling for socio-demographics. Results show that resources did not mediate the effects of stressors on the mental health outcomes. However, both higher depression and anxiety were associated with living closer to the care recipient (CR), less frequent visits, higher burden, being younger, being female, and less optimal income adequacy. In addition, higher depression was associated with lower use of coping strategies and higher education. Higher anxiety was also associated with lower levels of social support and higher familialism.

